# TCS-FEEL: Topology-Optimized Federated Edge Learning with Client Selection

**DOI:** 10.3390/s25216534

**Published:** 2025-10-23

**Authors:** Hui Chen, He Li

**Affiliations:** Department of Sciences and Informatics, Muroran Institute of Technology, Muroran 050-0071, Hokkaido, Japan; 23096002@muroran-it.ac.jp

**Keywords:** edge computing, federated learning, topology optimization, stochastic client selection, wireless networks

## Abstract

Federated learning (FL) enables distributed model training across sensor-equipped edge devices while preserving data privacy. However, its performance is often hindered by statistical heterogeneity among clients and system heterogeneity in dynamic wireless networks. To address these challenges, we propose TCS-FEEL, a topology-aware client selection framework that jointly considers user distribution, device-to-device (D2D) communication, and statistical similarity of client data. The proposed approach integrates randomized client sampling with an adaptive tree-based communication structure, where user devices not only participate in local model training but also serve as relays to exploit efficient D2D transmission. TCS-FEEL is particularly suited for sensor-driven edge intelligence scenarios such as autonomous driving, smart city monitoring, and the Industrial IoT, where real-time performance and efficient resource utilization are crucial. Extensive experiments on MNIST and CIFAR-10 under various non-IID data distributions and mobility settings demonstrated that TCS-FEEL consistently reduced the number of training rounds and shortened per-round wall-clock time compared with existing baselines while maintaining model accuracy. These results highlight that integrating topology control with client selection provides an effective solution for accelerating privacy-preserving and resource-efficient FL in dynamic, sensor-rich edge environments.

## 1. Introduction

The Internet of Things (IoT) is reshaping the landscape of global connectivity. With the increasing proliferation of interconnected IoT devices, numerous benefits are being introduced into everyday life. The IoT has been widely adopted in diverse domains such as smart homes, smart cities, the Industrial Internet of Things (IIoT) [1], and smart healthcare [2]. However, the explosive growth of IoT devices has posed significant challenges to conventional cloud computing paradigms, where vast volumes of raw data have to be transmitted to centralized servers for processing [3]. This approach has led to substantial latency, congestion, and scalability issues, undermining real-time responsiveness in latency-critical applications.

Edge computing addresses these limitations by processing data closer to their source, thereby significantly reducing latency [4]. This feature makes it particularly suitable for latency-sensitive IoT scenarios such as virtual reality, augmented reality [5], and autonomous driving [6]. In autonomous driving, for instance, real-time obstacle classification on sensor-equipped vehicles imposes stringent computational and communication requirements, necessitating highly efficient distributed intelligence at the network edge. Such edge devices, equipped with heterogeneous sensing and communication capabilities, further emphasize the need for scalable, secure, and resource-efficient distributed learning solutions.

When integrated with the IoT, edge computing offers multiple advantages, including high QoS, low latency, reduced energy consumption, and improved scalability [7]. However, it also introduces new challenges in terms of privacy and data security, as many devices collect sensitive personal or environmental information. Federated learning (FL) has emerged as a promising distributed intelligence paradigm in this context, enabling collaborative model training directly on devices without sharing raw sensor data [8]. This property is particularly critical in sensor-based systems such as autonomous vehicles, smart surveillance, and industrial monitoring, where privacy-preserving, real-time intelligence is essential.

Despite these advantages, FL performance in edge environments is often constrained by system heterogeneity (i.e., differences in computing, memory, and bandwidth capabilities) and statistical heterogeneity (i.e., non-identically and independently distributed data) [9]. System heterogeneity introduces straggler effects, where faster clients idle while waiting for slower ones, while statistical heterogeneity leads to biased global models and slower convergence. Moreover, traditional FL frameworks typically rely on star topologies, where direct device-to-server communication can incur high latency, particularly for resource-constrained or distant devices.

To mitigate these challenges, recent works have explored device-to-device (D2D) communication to complement device-to-server (D2S) links, thereby forming semi-decentralized FL architectures [10,11,12]. D2D communication can reduce energy consumption, enable localized model synchronization, and enhance fault tolerance in dynamic networks—characteristics that are essential to sensor-driven applications with mobile or intermittently connected nodes. Moreover, adaptive topology control further enhances robustness by accommodating device mobility and availability fluctuations [13].

On the other hand, client sampling strategies that jointly consider statistical and system heterogeneity have been developed to accelerate convergence [14]. Integrating such strategies with dynamic topology optimization is particularly critical to federated sensor networks, where both communication efficiency and learning performance need to be co-optimized.

**Figure 1:**
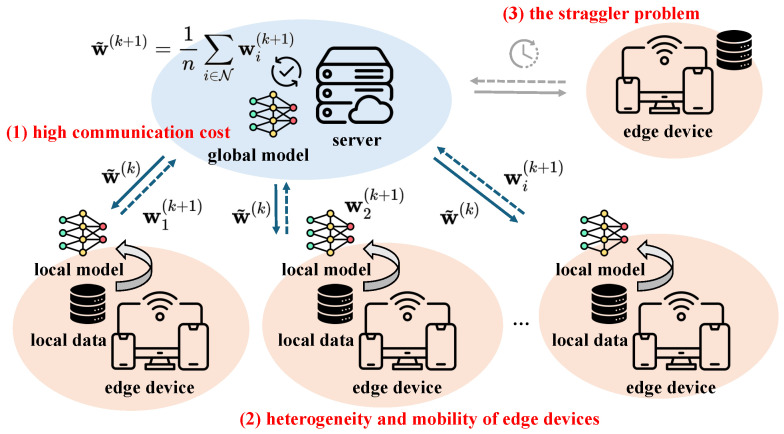
Main challenges in achieving communication-efficient FL in mobile edge environments, highlighted in red in the figure, include: (1) high communication cost arising from the frequent exchange of model parameters and increase in model size; (2) dynamic communication topology due to the heterogeneity and mobility of edge devices; (3) the straggler problem owing to the lack of joint optimization of communication and computation.

Figure 1 illustrates the main challenges in achieving communication-efficient FL in a general mobile edge environment. In this work, we address these challenges by introducing TCS-FEEL, a topology-aware client selection framework specifically designed for sensor-driven federated edge learning. By jointly optimizing communication topology and client selection, TCS-FEEL enables secure, low-latency, and resource-efficient collaborative intelligence directly at the sensing edge. This contribution closely aligns with the goals of sensor-based computational intelligence by facilitating privacy-preserving, adaptive, and scalable machine learning across distributed sensor networks.

For example, in autonomous driving scenarios, real-time obstacle classification tasks performed by on-vehicle edge devices involve substantial computational demand and require frequent model updates to adapt to rapidly changing environments. FL in such settings faces unique challenges, including dynamic topology changes resulting from high mobility and scenario-dependent non-IID data distributions. Therefore, the design of an efficient communication topology is essential to supporting such real-world applications, as it can significantly reduce communication latency, accelerate model updates, and ensure reliable coordination among mobile devices. The proposed TCS-FEEL framework is specifically designed to address these challenges by jointly optimizing communication topology and client selection, thereby enabling more efficient and robust FL in highly dynamic and heterogeneous environments.

In summary, the key contributions of this paper are as follows:

- We propose a topology-aware FL framework that leverages D2D communication to dynamically construct tree-based topologies, thereby reducing model transmission latency and improving communication efficiency. Furthermore, we develop a low-complexity algorithm to efficiently solve the topology optimization problem.
- We design a stochastic client selection strategy that evaluates client importance by jointly considering data volume, statistical distribution, and communication delay, effectively mitigating the adverse effects of non-IID data on convergence performance.
- We conduct extensive simulations in dynamic wireless edge networks with heterogeneous data distributions. The results demonstrate that the proposed TCS-FEEL framework achieves faster convergence and significantly lower communication time compared with state-of-the-art approaches.

The remainder of this paper is organized as follows: Section 2 reviews the related work. Section 3 introduces the system model, including the FL objective and the considered network model. Section 4 presents the proposed TCS-FEEL framework, formulates the optimization objective, and describes the integration of stochastic client selection and dynamic tree topology. Section 5 details the Monte Carlo-based Gradient Descent (MCGD) algorithm for solving the client selection problem. Section 6 presents the experimental setup and results, while Section 7 provides an in-depth discussion of the findings. Finally, Section 8 concludes the paper and outlines future research directions.

## 2. Related Work

The exploration and enhancement of communication efficiency in FL over dynamic edge wireless networks has become a rapidly growing research field. This section reviews existing studies, focusing on two major aspects: network topology designs and client sampling strategies for optimizing FL communication and convergence.

### 2.1. Network Topologies

In FL, the network topology defines how edge devices communicate with each other and ultimately with the central server. The highly dynamic link conditions in edge networks require FL frameworks to adopt adaptive and fault-tolerant topologies for model aggregation. Several survey papers provide comprehensive overviews of topology optimization in dynamic edge networks [13,15].

Hierarchical FL has demonstrated significant advantages in reducing communication overhead [16,17,18]. A tree topology is formed when additional layers are introduced between the server and devices, with the central server as the root node and edge devices as the leaf nodes. Zhang et al. [19] proposed a two-tier FL scheme where local D2D groups aggregate their results before transmitting them to the server, thereby reducing the communication overhead.

Huang et al. [20] further advanced hierarchical FL by enabling any device to serve as both an aggregation and relay node, thereby constructing a more flexible and adaptive tree topology. In their approach, devices with weak channel conditions to the server can offload their model updates to nearby devices with stronger connectivity, thereby mitigating high transmission delays. Moreover, by jointly optimizing both the communication topology and computation speed, their method employs a trained deep neural network (DNN) to infer the optimal topology dynamically.

Xu et al. [21] proposed a dynamic tree construction approach by leveraging D2D communication. Their method forms and optimizes a local communication graph in each round by using a minimum spanning tree algorithm, significantly reducing communication costs. However, this approach focuses solely on communication optimization and does not account for disparities in local training time, which limits its applicability in heterogeneous environments.

### 2.2. Client Sampling Strategies

Client selection plays a critical role in accelerating FL convergence. The total FL time depends on both the number of training rounds required to achieve the target accuracy and the wall-clock time per round [22].

Importance-based sampling has been widely adopted to mitigate the impact of statistical heterogeneity. In these approaches, clients are selected in each round based on their statistical characteristics, such as data quality and dataset size [23,24], or based on local gradient and loss information [25,26], thereby reducing the number of rounds needed to achieve the target accuracy.

Luo et al. [27] developed a stochastic sampling strategy that optimizes selection probabilities to jointly address both system and statistical heterogeneity, ensuring that the global model remains an unbiased weighted estimate of all local models while mitigating non-IID effects and maintaining fairness.

Chen et al. [28] proposed a latency-aware client selection framework that jointly optimizes client selection probabilities, the number of participating clients, and the total training rounds to minimize the overall latency of FL under data and system heterogeneity. Specifically, their method formulates a mixed-integer nonlinear optimization problem that incorporates both data size and communication delay and derives the optimal client participation probabilities by using grid search and polyhedral active set algorithms. However, this approach performs offline optimization based on static network conditions and does not adapt to time-varying topologies or dynamic communication states, limiting its applicability in highly mobile edge environments.

Xu et al. [21] further introduced a gradient-based client selection approach that assigns participation probabilities based on local gradient information. However, because this method requires all clients to complete local training before selection, it may exacerbate the impact of system heterogeneity.

While the above studies have advanced client selection strategies from different perspectives, most focus on either statistical heterogeneity or communication efficiency in isolation. This leaves an open problem of jointly considering both communication dynamics and system constraints in client selection, an aspect that motivates the design of the framework proposed in this paper.

## 3. System Model

This section presents the system model and key assumptions underlying the proposed TCS-FEEL framework. We consider a federated edge learning scenario consisting of a set of user devices (UDs) and a base station (BS) interconnected via device-to-server (D2S) and device-to-device (D2D) communication links. The proposed model is structured into three core components. Specifically, we first define the FL objective and client sampling mechanism, which govern distributed model training and aggregation across UDs. Next, we introduce the network model, which characterizes communication properties and captures topology dynamics under device mobility. Finally, we present the computation model, which quantifies local training time based on device processing capabilities. Key notations and definitions are summarized in Table 1.

### 3.1. Federated Learning Objective

We consider a collaborative learning environment that consists of a set of UDs indexed by {1,…,N} and a single BS indexed by {0}. We refer to all UDs and the BS collectively as *nodes*. Each UD i∈N is assumed to store a local dataset $D_{i}$ of size $\left|D_{i}\right|$. Let $x_{i}^{\left(t\right)}\in R^{d}$ and $y_{i}^{\left(t\right)}\in R^{l}$ denote the feature vector and label of the *t*-th sample on UD *i*, respectively, and let w∈W represent the model parameters belonging to the parameter space W.

We define the *loss function* $f\left(w,x_{i}^{\left(t\right)},y_{i}^{\left(t\right)}\right)$ as a function that quantifies the prediction error of a model parameterized by w on sample *t*. We define the *local loss* on UD *i* as the empirical mean of the sample-wise losses:

(1) $$F_{i}\left(w\right)≜\frac{1}{\left|D_{i}\right|}\sum_{t=1}^{\left|D_{i}\right|}f\left(w,x_{i}^{\left(t\right)},y_{i}^{\left(t\right)}\right).$$

We define a coefficient that quantifies the proportion of a local dataset size over the global dataset size as $p_{i}=\frac{\left|D_{i}\right|}{\sum_{j\in N}\left|D_{j}\right|},$ which denotes the relative weight of UD *i*’s data in the global dataset. To avoid bias toward UDs with smaller datasets, the FL framework minimizes the following weighted global loss:

(2) $$F\left(w\right)≜\sum_{i\in N}p_{i}\;F_{i}\left(w\right).$$

All UDs cooperate to learn a single global model $w^{★}$ that minimizes the global loss:

(3) $$w^{★}≜\arg\min_{w\in W}F\left(w\right).$$

Throughout this work, we assume a standard synchronous FL workflow, where each communication round consists of local training on the UDs followed by model aggregation at the BS.

### 3.2. Client Sampling

In each FL round, only a subset of UDs participate in training. Let $S_{k}\subseteq N$ denote the set of UDs selected in round *k*. UD *i* is independently included with probability $\varphi_{i}^{\left(k\right)}\in \left(0,1\right]$. The sampling process is repeated until $S_{k}$ becomes a non-empty subset. Let

$$A_{k}≜\left\{S_{k}\neq ⌀\right\},\;P\left(A_{k}\right)=1-\prod_{j\in [N]}\left(1-\varphi_{j}^{\left(k\right)}\right).$$

The conditional inclusion probability of UD *i* is given by

(4) $$q_{i}^{\left(k\right)}\;≜\;P\left(i\in S_{k}\;|\;A_{k}\right)\;=\;\frac{\varphi_{i}^{\left(k\right)}}{1-\prod_{j\in [N]}\left(1-\varphi_{j}^{\left(k\right)}\right)}.$$

Let ${\tilde{w}}^{\left(kE\right)}$ denote the global model available to the UDs at the start of round *k*, and let $w_{i}^{\left((k+1)E\right)}$ denote UD *i*’s local model after *E* local epochs in round *k* (initialized from ${\tilde{w}}^{\left(kE\right)}$). The BS updates the global model via inverse-probability weighting:

(5) $${\tilde{w}}^{\left((k+1)E\right)}\;=\;{\tilde{w}}^{\left(kE\right)}\;+\;\sum_{i\in S_{k}}\frac{p_{i}}{\;q_{i}^{\left(k\right)}\;}\;\left(\;w_{i}^{\left((k+1)E\right)}-{\tilde{w}}^{\left(kE\right)}\right).$$

The factor $p_{i}/q_{i}^{\left(k\right)}$ ensures unbiasedness with respect to client selection, i.e.,

(6) $$E\;\left[\;\sum_{i\in S_{k}}\frac{p_{i}}{q_{i}^{\left(k\right)}}\left(w_{i}^{\left((k+1)E\right)}-{\tilde{w}}^{\left(kE\right)}\right)\;|\;A_{k}\right]=\sum_{i\in [N]}p_{i}\left(w_{i}^{\left((k+1)E\right)}-{\tilde{w}}^{\left(kE\right)}\right).$$

### 3.3. Network Model

We consider two communication modes: (i) access links between UDs and the BS, comprising uplink and downlink transmissions, and (ii) D2D links between nearby UDs. To improve reliability and efficiency, we adopt dedicated bandwidth for each mode: $\Phi_{D2D}$ Hz for D2D, $\Phi_{\mathrm{UL}}$ Hz for uplink, and $\Phi_{\mathrm{DL}}$ Hz for downlink. This orthogonal allocation prevents bandwidth contention across communication types and stabilizes channel conditions.

Directed link rates are estimated in each FL round via periodic probe packets. Let $\gamma_{i,j}^{\left(k\right)}$ denote the measured average throughput of directed link (i,j) in round *k* (including D2D links (i,j), uplinks (i,0), and downlinks (0,i)). The BS collects these measurements from UDs and maintains global knowledge of $\left\{\gamma_{i,j}^{\left(k\right)}\right\}$.

At round *k*, the network is modeled as a weighted directed graph

$$G^{\left(k\right)}≜(V,\;E^{\left(k\right)}),$$

where V represents the set of nodes (i.e., the UDs and the BS) and a directed edge $\left(i,j\right)\;\in \;E^{\left(k\right)}$ is assigned the latency weight

(7) $$l^{\left(k\right)}\left(i,j\right)≜\frac{M}{\gamma_{i,j}^{\left(k\right)}},$$

with *M* denoting the model size in bits. We define a directed path from node *i* to *j* in round *k* as a finite ordered sequence of vertices:

(8) $$L_{i\to j}^{\left(k\right)}≜{\left(v_{r}\right)}_{r=0}^{m},\;v_{0}=i,\;v_{m}=j,\;\left(v_{r-1},v_{r}\right)\in E^{\left(k\right)}\;\;\forall r\in \left\{1,\ldots ,m\right\}.$$

Therefore, the transmission time equals the sum of edge weights:

(9) $$T^{\left(k\right)}\;\left(L_{i\to j}^{\left(k\right)}\right)=\sum_{r=1}^{m}l^{\left(k\right)}\left(v_{r-1},v_{r}\right)=\sum_{r=1}^{m}\frac{M}{\gamma_{v_{r-1},v_{r}}^{\left(k\right)}}.$$

Each UD $i\in S_{k}$ downloads and uploads the model along the shortest directed paths in $G^{\left(k\right)}$. In case multiple shortest paths exist, ties are broken deterministically. First, paths with fewer hops are preferred. If a tie remains, the lexicographically smallest next hop is selected. Under this rule, each node has a unique parent, and the selected paths form a tree topology rooted in the BS.

### 3.4. Computation Model

In round *k*, each UD $i\in S_{k}$ performs *E* local epochs of mini-batch stochastic gradient descent (SGD) on its dataset $D_{i}$. Let $\tau_{i}^{\left(k\right)}$ denote the average processing time per sample on UD *i* in round *k*. The local training time is

(10) $$T_{i,\mathrm{train}}^{\left(k\right)}=E\;\left|D_{i}\right|\;\;\tau_{i}^{\left(k\right)}.$$

## 4. Our Framework

Building on the system model defined in Section 3, we present the design of the proposed TCS-FEEL framework, which aims to minimize the total wall-clock training time by jointly leveraging the latency and computation models as optimization variables. Specifically, the network latency model guides dynamic topology construction, while the computation model informs client selection strategies.

This section is organized as follows: First, we provide an overview of the overall workflow of TCS-FEEL, outlining the key stages of the training and communication process. Next, we describe the optimization of communication topology, which exploits D2D relaying to reduce transmission latency and improve network efficiency. Then, we present the formulation of client selection criteria, which jointly consider system and statistical heterogeneity to accelerate convergence. Finally, we define the overall optimization objective of TCS-FEEL, aiming to minimize the total training time while maintaining high model accuracy.

### 4.1. Workflow Overview

**Figure 2:**
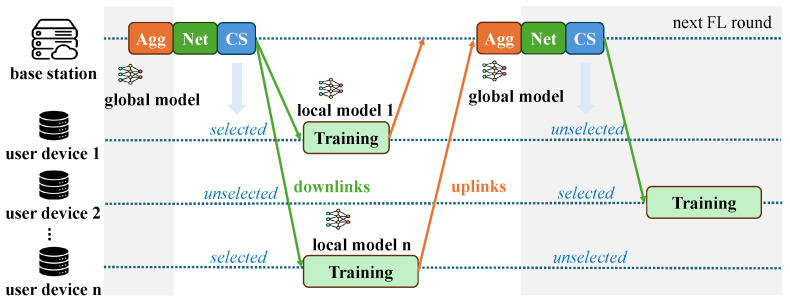
Overall workflow of the proposed TCS-FEEL framework. As illustrated, each round consists of network topology optimization (Net), client selection (CS), local training, and model aggregation (Agg).

Figure 2 illustrates the overall workflow of the proposed TCS-FEEL framework. In each FL round, the system dynamically computes the shortest communication paths in real time based on the current network topology and channel conditions, thereby ensuring that routing decisions adapt to mobility and link variations. The overall process is summarized as follows:

1. The system first performs real-time topology construction by computing the shortest upload and download paths for all UDs based on the current network state. It then calculates their selection probabilities by jointly considering transmission latency, dataset size, and data distribution similarity.
2. A subset of UDs is sampled according to these probabilities to participate in the current round, with the constraint that at least one UD is selected.
3. The global model maintained at the BS is transmitted to the selected UDs along the shortest download paths.
4. Each selected UD performs local model training on its local dataset.
5. The locally updated models are uploaded to the BS along the shortest upload paths. Since the network topology may change during local training due to device mobility, the shortest paths are recomputed in real time before the upload phase. If a selected UD also acts as a relay, it aggregates received updates with its own before forwarding.
6. Finally, the BS aggregates all received updates to generate the new global model, which is distributed in the next round.

### 4.2. Network Topology Optimization

Efficient communication topology construction is essential to minimizing transmission latency in federated edge learning, particularly under dynamic network conditions caused by device mobility. In the proposed TCS-FEEL framework, the communication topology is dynamically optimized in each FL round to establish low-latency routes between UDs and the BS by leveraging D2D relaying opportunities.

At time instant *t*, the network environment is modeled as a weighted directed graph $G^{\left(t\right)}=\left(V,E^{\left(t\right)},\ell^{\left(t\right)}\right)$, where V denotes the set of nodes (UDs and the BS), $E^{\left(t\right)}$ represents the set of directed links, and $\ell^{\left(t\right)}\left(u,v\right)>0$ denotes the latency weight of edge (u,v). For each selected UD $i\in S_{t}$, two routing problems must be solved: (i) the uplink problem, which seeks the shortest directed path from UD *i* to the BS (0), and (ii) the downlink problem, which seeks the shortest directed path from the BS (0) to UD *i*.

These routing tasks are formulated as classical single-source or single-destination shortest-path problems on a non-negatively weighted directed graph. Therefore, we employ the Dijkstra algorithm, which guarantees the optimal solution in polynomial time and has been widely used in network optimization [29,30,31]. The shortest-path computation is highly efficient and incurs negligible overhead relative to the total round duration. Once the shortest paths for all participating UDs are determined, the resulting routing structure naturally forms a tree topology rooted at the BS, enabling multi-hop model uploading and downloading while minimizing per-round communication delay.

Let *s* be the source and *g* the target. The Dijkstra algorithm maintains, for each node *v*, a tentative distance d(v), a hop count h(v), and a predecessor pred(v). A min-priority queue is keyed lexicographically by the tuple $\left(d(v),\;h(v),\;v\right)$ to obtain deterministic results: first minimize distance; if equal, prefer fewer hops; if still equal, use the node index to break ties.

For uploads, we set (s,g)=(i,0) and run the Dijkstra algorithm on $G^{\left(t\right)}$ for each i∈N or equivalently perform a single Dijkstra run from 0 on the reverse graph $G_{\mathrm{rev}}^{\left(t\right)}=\left(V,E_{\mathrm{rev}}^{\left(t\right)},\ell^{\left(t\right)}\right)$ with $E_{\mathrm{rev}}^{\left(t\right)}=\left\{\left(v,u\right):\left(u,v\right)\in E^{\left(t\right)}\right\}$ to obtain all i→0 distances $d^{\left(t\right)}\left(i,0\right)$ and next hops simultaneously. For downloads, we perform one Dijkstra run from s=0 on $G^{\left(t\right)}$ to recover every 0→i path.

Let $P_{i\to 0}^{\left(t\right)}$ ($Q_{0\to i}^{\left(t\right)}$) denote the selected shortest upload (download, respectively) path. We define the routing function $r_{t}\left(v\right)$ as the next hop toward the BS on $P_{i\to 0}^{\left(t\right)}$. The predecessor map returned by the Dijkstra algorithm induces a BS-rooted shortest-path tree for uploads (and, analogously, for downloads). Algorithm 1 computes the shortest upload path $P_{i\to 0}^{\left(t\right)}$ for every UD *i* and the corresponding minimum transmission time:

(11) $$T_{↑}^{\left(t\right)}\left(i\right)=\sum_{\left(u,v\right)\in P_{i\to 0}^{\left(t\right)}}\ell^{\left(t\right)}\left(u,v\right).$$

The shortest download paths and their times are obtained analogously by running the Dijkstra algorithm from the BS on $G^{\left(t\right)}$:

(12) $$T_{↓}^{\left(t\right)}\left(i\right)\;=\;\sum_{\left(u,v\right)\in Q_{0\to i}^{\left(t\right)}}\ell^{\left(t\right)}\left(u,v\right).$$

With a binary heap, the Dijkstra algorithm runs in $O\;\left((|V|+|E^{\left(t\right)}|)\log|V|\right)$ time per execution. Equivalently, performing a single Dijkstra run from the BS on the reverse graph yields all i→0 upload shortest paths within the same bound per round.
**Algorithm 1** Dijkstra-based upload routing in time instant *t*.**Require:** Directed graph $G^{\left(t\right)}=\left(V,E^{\left(t\right)},\ell^{\left(t\right)}\right)$ with $\ell^{\left(t\right)}\left(u,v\right)>0$; UD index set N={1,…,N}; BS index 0**Ensure:** 
For each i∈N: shortest upload path $P_{i\to 0}^{\left(t\right)}$ and time $T_{↑}^{\left(t\right)}\left(i\right)$ 1:**Build in-neighbor adjacency:** for each $\left(x,y\right)\in E^{\left(t\right)}$, append *x* to InNbr(y) and store $\ell^{\left(t\right)}\left(x,y\right)$ 2:**Initialize:** for all v∈V, set d(v)←+∞, pred(v)←⌀; set d(0)←0 3:Initialize a min-priority queue *Q* keyed by d(·) and insert 0 4:**while** *Q* not empty **do** 5:    u←Q.ExtractMin() 6:    **for all** v∈InNbr(u) **do** 7:        $d^{\prime }\leftarrow d\left(u\right)+\ell^{\left(t\right)}\left(v,u\right)$ 8:        **if** $d^{\prime }<d\left(v\right)$ **then** 9:           $d\left(v\right)\leftarrow d^{\prime }$, pred(v)←u  10:           **if** v∈Q **then**  11:               Q.DecreaseKey(v)  12:           **else**  13:               Q.Insert(v)  14:           **end if**  15:        **end if**  16:    **end for**  17:**end while**  18:**Output per UD**  19:**for all** i∈N **do**  20:    **if** d(i)=+∞ **then**  21:        $P_{i\to 0}^{\left(t\right)}\leftarrow \left[\;\right]$; $T_{↑}^{\left(t\right)}\left(i\right)\leftarrow +\infty$  22:    **else**  23:        path←[i], v←i  24:        **while** v≠0 **do**  25:           v←pred(v); **if** v=⌀ **then** path←[]; d(i)←+∞; **break**  26:           append *v* to path  27:        **end while**  28:        $P_{i\to 0}^{\left(t\right)}\leftarrow \mathrm{path}$; $T_{↑}^{\left(t\right)}\left(i\right)\leftarrow d\left(i\right)$  29:    **end if**  30:**end for**  31:**return** $\left\{\;\left(P_{i\to 0}^{\left(t\right)},\;T_{↑}^{\left(t\right)}\left(i\right)\right)\;:\;i\in N\;\right\}$

### 4.3. Client Selection Strategy

Let the expected number of participating UDs per round be *K*. To improve training efficiency and reduce the overall wall-clock time, UDs with lower transmission latency, larger local dataset sizes, and data distributions closer to the global distribution are assigned higher participation probabilities. The total time for UD *i* in round *k* is expressed as

(13) $$T_{i,\mathrm{total}}^{\left(k\right)}=T_{↓}^{\left(k\right)}\left(i\right)+T_{i,\mathrm{train}}^{\left(k\right)}+T_{↑}^{\left(k\right)}\left(i\right),$$

where $T_{↓}^{\left(k\right)}\left(i\right)$ and $T_{↑}^{\left(k\right)}\left(i\right)$ represent the model download and upload transmission times, respectively. Importantly, both terms are estimated during the client selection stage, i.e., prior to the start of round *k*, based on the current network topology and channel conditions. This enables the server to anticipate the communication cost of each candidate UD before selection, thereby facilitating informed decision making that jointly accounts for communication delay, computation time, and statistical contribution.

We define a normalized latency factor, where a smaller $T_{i,\mathrm{total}}^{\left(k\right)}$ corresponds to a larger $V_{i}$:

(14) $$V_{i}\;≜\;\frac{1/T_{i,\mathrm{total}}^{\left(k\right)}}{\max_{j\in N}\;1/T_{j,\mathrm{total}}^{\left(k\right)}}\;\in \;\left(0,1\right],$$

The normalized dataset size is given by

(15) $$D_{i}\;≜\;\frac{|D_{i}|}{\sum_{j\in N}\left|D_{j}\right|}\;\in \;\left(0,1\right].$$

Let $P_{g}$ be the global data distribution. We quantify the mismatch between the local distribution $P_{i}$ and $P_{g}$ by using the *Jensen–Shannon (JS) divergence*, which is defined as a symmetric and bounded measure derived from the Kullback–Leibler divergence:

(16) $$\mathrm{JSD}\;\left(P_{i}\;∥\;P_{g}\right)=\frac{1}{2}\;\mathrm{KL}\;\left(P_{i}\;∥\;M\right)+\frac{1}{2}\;\mathrm{KL}\;\left(P_{g}\;∥\;M\right),$$

where $M=\frac{1}{2}\;\left(P_{i}+P_{g}\right)$. For discrete labels C, this can be expressed as

(17) $$\mathrm{JSD}\;\left(P_{i}\;∥\;P_{g}\right)=\frac{1}{2}\sum_{c\in C}P_{i}\left(c\right)\;\log\;\frac{P_{i}\left(c\right)}{M(c)}+\frac{1}{2}\sum_{c\in C}P_{g}\left(c\right)\;\log\;\frac{P_{g}\left(c\right)}{M(c)}.$$

We then map this divergence to a similarity score:

(18) $$S_{i}\;≜\;\exp\;\left(-\lambda \;\mathrm{JSD}\;\left(P_{i}\;∥\;P_{g}\right)\right)\;\in \;\left(0,1\right],$$

where λ>0 is a scaling coefficient that controls the sensitivity of the similarity score to distribution mismatch.

Let $u_{i}\in R$ denote the utility score of client *i*, which is designed to increase with lower total delay, larger dataset size, and higher similarity between the local and global data distributions. We define $u_{i}$ as

(19) $$u_{i}\;≜\;\alpha_{V}\log V_{i}+\alpha_{D}\log D_{i}+\alpha_{S}\log S_{i},$$

where $\alpha_{V},\alpha_{D},\alpha_{S}\geq 0$ are weighting coefficients that control the relative importance of the three factors.

### 4.4. Optimization Objective

Given an expected number *K* of participating clients per round, our goal is to determine a probability distribution ${\left\{\varphi_{i}^{\left(k\right)}\right\}}_{i\in N}$ that maximizes the expected utility of the selected clients while maintaining a certain level of randomness in the selection process. To formalize the trade-off between utility maximization and probability dispersion, we formulate the following entropy-regularized optimization problem:

(20) $$\begin{array}{cc} \max_{\left\{\varphi_{i}\right\}}\; & \sum_{i\in N}u_{i}\varphi_{i}\;-\;\beta \sum_{i\in N}\varphi_{i}\log\varphi_{i}\\ s.t.\;\; & \sum_{i\in N}\varphi_{i}=K,\\ \; & 0\leq \varphi_{i}\leq 1,\;\forall i\in N, \end{array}$$

where β>0 is a temperature coefficient that controls the trade-off between exploitation of high-utility clients and exploration across all clients.

The first term in (20) encourages assigning higher probabilities to clients with larger $u_{i}$ values, while the second term serves as an entropy regularizer to avoid deterministic or overly skewed selections. The constraint $\sum_{i}\varphi_{i}=K$ ensures that the expected number of participating clients per round equals *K*.

By applying the method of Lagrange multipliers, the optimal solution is obtained in closed form as

(21) $$\varphi_{i}^{\left(k\right)}=\frac{K\;\exp\;\left(u_{i}/\beta \right)}{\sum_{j\in N}\exp\;\left(u_{j}/\beta \right)}.$$

This allocation ensures that clients with smaller delay, larger dataset size, and more globally representative data distributions have a higher probability of being selected.

## 5. Monte Carlo-Based Gradient Descent for Client Selection

In our design, the utility scores of clients dynamically vary across rounds due to changes in network conditions. To efficiently solve the optimization problem defined in Equation (20), we employ a low-complexity Monte Carlo-Based Gradient Descent (MCGD) approach. The detailed procedure is summarized in Algorithm 2, which iteratively updates the selection probabilities based on sampled gradients and converges to an optimal client selection strategy with low computational overhead.
**Algorithm 2** MCGD for client selection in round *k*.**Require:** 
*K*, β>0, step sizes ${\left\{\eta_{t}\right\}}_{t=0}^{T-1}$, inner iterations *T*, mini-batch size *B* 1:Measure $V_{i}^{\left(k\right)},D_{i}^{\left(k\right)},S_{i}^{\left(k\right)}$ for all clients and compute $u_{i}^{\left(k\right)}$ 2:Initialize $\varphi^{\left(0\right)}$ (e.g., softmax solution $\propto \exp(u_{i}^{\left(k\right)}/\beta )$), then project: $\varphi^{\left(0\right)}\leftarrow \Pi_{C}\left[\varphi^{\left(0\right)}\right]$ 3:**for** t=0 to T−1 **do** 4:    Sample mini-batch $B_{t}={\left\{\xi_{b}\right\}}_{b=1}^{B}$ 5:    Compute gradient estimates:g^i(t)=1B∑b=1Bui(ξb)−β1+logφi(t) 6:    Gradient ascent step:φ˜(t+1)=φ(t)+ηtg^(t) 7:    Project back to feasible set:$$\varphi^{\left(t+1\right)}\leftarrow \Pi_{C}\left[{\tilde{\varphi }}^{\left(t+1\right)}\right]$$ 8:**end for** 9:**return** $\varphi^{\left(k\right)}\leftarrow \varphi^{\left(T\right)}$

Given the client utility score $u_{i}=\alpha_{V}\log V_{i}+\alpha_{D}\log D_{i}+\alpha_{S}\log S_{i}$, the entropy-regularized objective in round *k* is defined as

(22) $$F^{\left(k\right)}\left(\varphi \right)=\sum_{i\in N}u_{i}^{\left(k\right)}\;\varphi_{i}-\beta \sum_{i\in N}\varphi_{i}\log\varphi_{i},\;s.t.\;\;\sum_{i}\varphi_{i}=K,\;0<\varphi_{i}\leq 1,$$

where β>0 controls the trade-off between utility maximization and probability dispersion and *K* denotes the expected number of selected clients per round. When $u_{i}^{\left(k\right)}$ is computed from stochastic measurements (e.g., probe-based throughput or empirical data distribution), we denote $u_{i}^{\left(k\right)}=u_{i}\left(\xi^{\left(k\right)}\right)$, where $\xi^{\left(k\right)}$ represents the random state in round *k*. The optimization target then becomes

(23) $$\max_{\varphi }\;E_{\xi^{\left(k\right)}}\left[F^{\left(k\right)}\left(\varphi ;\xi^{\left(k\right)}\right)\right].$$

The partial derivative with respect to $\varphi_{i}$ is

(24) $$\nabla_{i}F^{\left(k\right)}\left(\varphi ;\xi \right)=u_{i}\left(\xi \right)-\beta \left(1+\log\varphi_{i}\right),$$

and a Monte Carlo mini-batch unbiased estimator over a batch B is given by

(25) g^i=1|B|∑ξ∈Bui(ξ)−β1+logφi.

Let C denote the feasible set,

(26) $$C≜\left\{\varphi \in R^{N}\;|\;\sum_{i}\varphi_{i}=K,\;0<\varphi_{i}\leq 1\right\}$$

and let $\Pi_{C}\left[\cdot \right]$ represent the Euclidean projection onto C, which can be implemented via capped-simplex water filling (clipping and renormalization).

A projected softmax initialization $\varphi_{i}\propto \exp\left(u_{i}^{\left(k\right)}/\beta \right)$ is adopted to provide an effective warm start, which consistently accelerates convergence in practice. Step sizes follow a diminishing schedule (e.g., $\eta_{t}=\eta_{0}/\sqrt{t+1}$), guaranteeing convergence under standard stochastic approximation theory. The projection operator $\Pi_{C}$ is implemented via a capped-simplex water-filling algorithm with O(NlogN) complexity, while each gradient update step after aggregation requires O(N) time. Because $u_{i}^{\left(k\right)}$ varies in every round, the algorithm is executed at each FL round to ensure that the selection probabilities $\left\{\varphi_{i}^{\left(k\right)}\right\}$ are continuously and optimally adapted to the prevailing network and data conditions.

## 6. Evaluation

This section evaluates the performance of the proposed TCS-FEEL framework through extensive simulations. We first describe the experimental setup, including datasets, model configurations, network parameters, and baseline schemes. Next, we present results on key performance metrics such as per-round latency, convergence speed, communication energy consumption, and topology dynamics.

### 6.1. Experimental Setup

**Figure 3:**
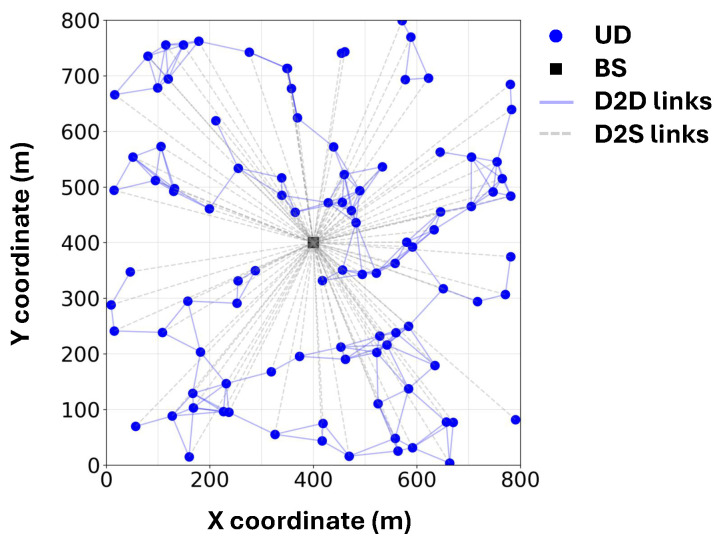
An example of the simulated system.

An illustrative example of the simulated system layout is shown in Figure 3. The values of the parameters that we used for simulations are summarized in Table 2.

**UD distribution and mobility:** We simulate the FL process within an 800×800 m^2^ square area, where a single BS is deployed at the center and 100 UDs are uniformly distributed across the area. The Random Direction Mobility Model [32] is adopted to emulate the mobility of UDs. Specifically, at each second, every UD moves in a randomly chosen direction with an average speed of *v* m/s. We assume that two UDs can establish a D2D link if their distance does not exceed 100 m, which reflects a realistic upper bound for D2D connectivity in urban and vehicular scenarios [21,33].

**Network:** Network probes are simulated by applying Shannon’s capacity formula under Rayleigh fading for each D2D, uplink, and downlink connection, given the distance and channel conditions at each time snapshot. The throughput of D2D links, uplinks, and downlinks is modeled using Shannon’s formula:

(27) $$\gamma_{i,j}=C\;\Phi_{i,j}\log\;\left(1+\frac{|h_{i,j}|\;\pi_{i}\;d_{i,j}^{-\zeta }}{n_{0}}\right),$$

where *C* is the channel capacity utilization rate, $\pi_{i}$ is the transmission power of node *i*, $d_{i,j}$ denotes the Euclidean distance between nodes *i* and *j*, ζ is the path-loss exponent, and $n_{0}$ represents the background noise. The channel gain $|h_{i,j}|$ follows a zero-mean unit-variance Rayleigh distribution when UD *i* transmits to UD *j*. We assume orthogonal resource allocation among D2D, uplink, and downlink links based on a TDMA scheme, following widely adopted modeling practices in the D2D communication literature [33]. Specifically, we set $\Phi_{i,j}=\Phi_{D2D}$ when both *i* and *j* are UDs, $\Phi_{i,j}=\Phi_{\mathrm{UL}}$ when *j* is the BS, and $\Phi_{i,j}=\Phi_{\mathrm{DL}}$ when *i* is the BS. This orthogonality assumption simplifies interference modeling and enables a focused analysis of the impact of topology optimization and client selection on system performance. However, we acknowledge that this stylized assumption neglects potential co-channel interference effects present in real-world deployment, which may lead to slightly optimistic estimates of achievable throughput.

**Model and datasets:** Each UD trains a ResNet-9 architecture consisting of four convolutional blocks and two residual blocks, followed by a global average pooling layer and a fully connected output layer. The input channels are set to one for MNIST and three for CIFAR-10, with 10 output classes. The model contains approximately 6.57 million parameters and a total size of 25.09 Mb.

We adopt stochastic gradient descent (SGD) as the local optimizer, with a momentum of 0.5 and a weight decay of $5\times 10^{-4}$. The initial learning rate is set to 0.01 and decayed following a cosine annealing schedule. Each client trains its local model for E=5 epochs per round using a batch size of 64 and a cross-entropy loss function.

We adopted the widely used MNIST and CIFAR-10 datasets for evaluation. The MNIST dataset consists of grayscale images of size 28×28 across 10 classes. The CIFAR-10 dataset contains color images of size 32×32×3 across 10 classes. To simulate varying degrees of data heterogeneity, we partition the training data across UDs using a Dirichlet distribution [34]. A smaller value of α (e.g., α=0.05) leads to highly skewed data distributions, where each UD may only possess samples from a limited number of classes. In contrast, larger values of α (e.g., α=1) produce more balanced data distributions across UDs, thereby reducing the degree of non-IID.

**Hardware and implementation details:** All experiments were conducted on a workstation equipped with an Intel^®^ Core^TM^ i9-10850K CPU running at 3.60 GHz with 20 logical cores, 32 GB RAM, and the Ubuntu 20.04.4 LTS (64-bit) operating system. All programs were implemented in Python 3.10 using the NumPy and NetworkX libraries. To ensure consistent single-threaded execution and accurate measurement of the control-plane overhead, all tests were run with OMP_NUM_THREADS=1 and MKL_NUM_THREADS=1.

Each experiment was repeated five times with different random seeds controlling data partitioning, client sampling, model initialization, and mobility patterns. All reported improvements were statistically validated using pair-wise Mann–Whitney *U* tests between TCS-FEEL and other baseline methods across five independent runs. Statistical significance is denoted as p<0.05 and p<0.01.

**Wall-clock time measurements:** The total per-round wall-clock time consists of two components: (i) local computation time, which is proportional to the number of local epochs and the size of the local dataset, and (ii) communication time, including model uploading and potential D2D relaying. Fixing E=5 enables us to isolate the effects of topology optimization and client selection on communication latency and overall training time.

**Baselines:** We compare TCS-FEEL with three baseline policies under different scenarios, described as follows:

- **FedAvg (Star) [35]:** In this scheme, a subset of UDs is randomly selected in each round, where each UD is chosen with probability 0.1. The selected UDs directly send their local models to the BS for aggregation, following a star topology.
- **CSTAR [21]:** In CSTAR, the selection probability of each UD in each round is determined by both latency and the contribution of its model update. Different from our TCS-FEEL, only the selected UDs participate in model transmission, while unselected UDs do not take part in training or forwarding.
- **LACS (Latency-Aware Client Selection) [28]:** In this method, client selection is formulated as an offline optimization problem to minimize overall training latency under data and system heterogeneity. Unlike our TCS-FEEL, LACS assumes a static star network. The optimal selection probabilities are obtained via grid search and polyhedral active set algorithms based on the initial network conditions. For a fair comparison, we compute the latency in LACS using the initial communication parameters of the network.
- **FedAvg (Tree):** The client selection strategy is the same as FedAvg (Star), where each UD is randomly selected with probability 0.1 in each round. Meanwhile, the model transmission is consistent with our TCS-FEEL, where all UDs in the system are used to construct a dynamic tree topology.
- **TCS-FEEL (Star):** This variant applies the proposed stochastic client selection strategy while maintaining a static star topology for communication, where all selected UDs transmit their local models directly to the BS without D2D relaying.

### 6.2. Model Convergence Evaluation

We compared TCS-FEEL with baseline schemes on the CIFAR-10 and MNIST datasets in terms of model convergence speed. Specifically, we measured the validation accuracy of the aggregated model, as well as the number of rounds required to reach target accuracies on the two datasets under different non-IID settings.

**Figure 4:**
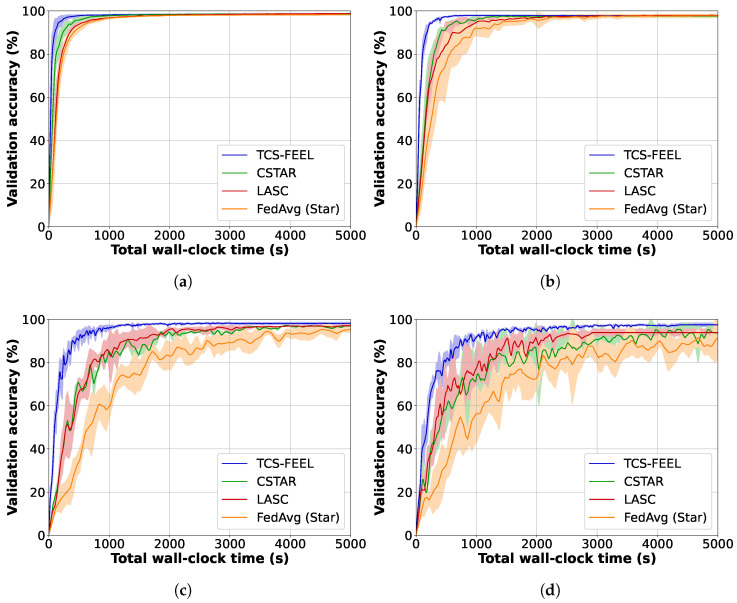
Model convergence speed and wall-clock training time for our method and baseline schemes on MNIST dataset under different non-IID settings (runs n=5): (**a**) α=0.5, (**b**) α=0.1, (**c**) α=0.05, and (**d**) α=0.03.

Figure 4 illustrates the global model accuracy achieved by different FL schemes on the MNIST dataset under different non-IID settings. The curves represent the average validation accuracy, while the shaded areas denote the standard deviation across multiple runs. Table 3 shows the number of rounds required to achieve a validation accuracy level of 95% on MNIST under different non-IID settings. Obviously, our TCS-FEEL consistently outperforms all baseline schemes across all scenarios. Specifically, when α=0.1, TCS-FEEL reduces the number of convergence rounds by 28.2% compared with CSTAR and by more than 42.0% compared with LASC and FedAvg (Star). When α=0.05, TCS-FEEL reduces the number of convergence rounds by 29.1% compared with CSTAR and by more than 45.9% compared with LASC and FedAvg (Star) (p<0.05). When α=0.03, TCS-FEEL requires 11.1% fewer convergence rounds than CSTAR and over 21.7% fewer than both LASC and FedAvg (Star) (p<0.05).

**Figure 5:**
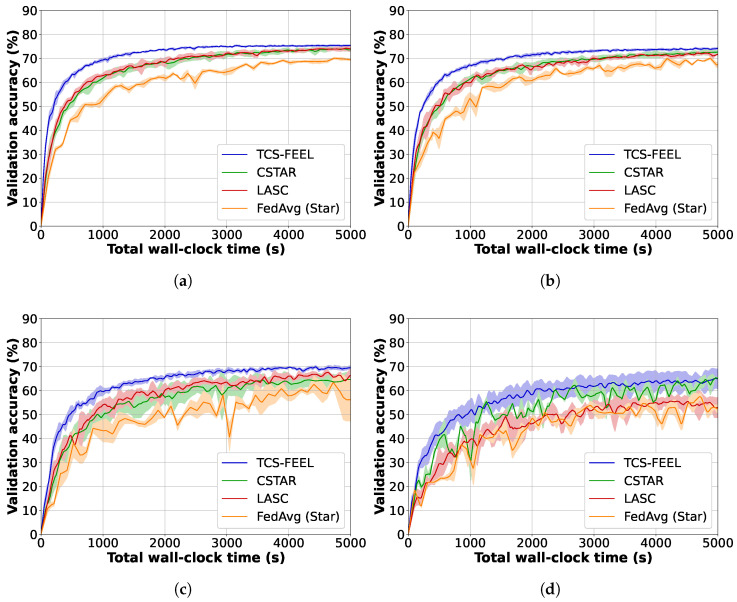
Model convergence speed and wall-clock training time for our method and baseline schemes on CIFAR-10 dataset under different non-IID settings (runs n=5): (**a**) α=0.5, (**b**) α=0.3, (**c**) α=0.1, and (**d**) α=0.05.

Similarly, Figure 5 shows the global model accuracy achieved by different FL schemes on the CIFAR-10 dataset under different non-IID settings. The curves represent the average validation accuracy, while the shaded areas denote the standard deviation across multiple runs. Table 4 shows the number of rounds required to achieve a validation accuracy level of 70% on CIFAR-10 under different non-IID settings. The results show that TCS-FEEL converges in fewer rounds than the random selection baseline. Specifically, when α=0.5, TCS-FEEL reduces the number of convergence rounds by more than 25.4% compared with FedAvg (Star) (p<0.05). When α=0.3, TCS-FEEL achieves over 29.7% fewer convergence rounds than FedAvg (Star) (p<0.05). When α=0.1, TCS-FEEL achieves more than 49.7% fewer convergence rounds than FedAvg (Star) (p<0.01). The model did not reach 70% accuracy on all methods when α=0.05.

### 6.3. Per-Round Latency Analysis

**Figure 6:**
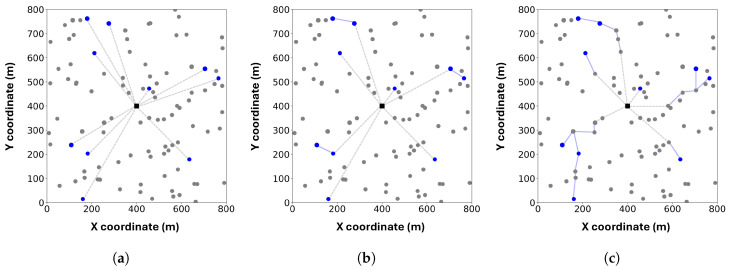
Visualization of three topologies: (**a**) Star, (**b**) CSTAR, (**c**) TCS-FEEL. Gray circles represent unselected UDs, and blue circles represent selected UDs. Gray dashed lines denote D2S links, and blue solid lines denote D2D links.

To provide an intuitive understanding of the different communication structures, we visualize the topologies of FedAvg (Star), CSTAR, and TCS-FEEL in Figure 6. In the star topology adopted by FedAvg, all selected UDs communicate directly with the BS, which often creates a communication bottleneck and leads to higher latency, especially in dense networks. In contrast, the tree-based topology leverages D2D communication, allowing UDs to relay model updates through intermediate devices. Compared with CSTAR, which relies only on those selected UDs for transmission, our TCS-FEEL further enhances efficiency by effectively utilizing the available D2D communication opportunities. As a result, TCS-FEEL achieves shorter time per round, leading to faster convergence and lower latency in dynamic edge environments.

**Figure 7:**
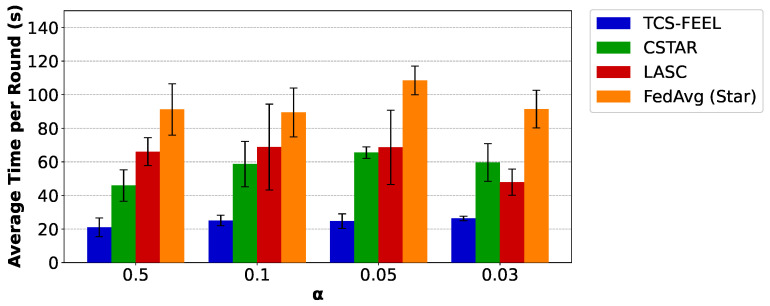
The average time per round for our method and baseline schemes on the MNIST dataset under different non-IID settings (runs n=5, mean ± std).

Next, we compare the average per-round latency of TCS-FEEL with the baseline schemes. Figure 7 illustrates the average time per round required by different FL methods on the MNIST dataset under varying degrees of non-IID data distributions. Specifically, when α=0.5, the proposed tree-based topology reduces the per-round latency by 54.3% compared with CSTAR, by 68.2% compared with LASC (p<0.05), and by 77.0% compared with FedAvg (Star) (p<0.01). When α=0.1, TCS-FEEL reduces the per-round latency by 57.3% compared with CSTAR, by 63.6% compared with LASC, and by 72.0% compared with FedAvg (Star) (p<0.01). When α=0.05, TCS-FEEL reduces the per-round latency by 62.4% compared with CSTAR (p<0.05), by 64.1% compared with LASC, and by 77.3% compared with FedAvg (Star) (p<0.01). When α=0.03, TCS-FEEL reduces the per-round latency by 55.9% compared with CSTAR, by 45.1% compared with LASC, and by 71.3% compared with FedAvg (Star) (p<0.01).

**Figure 8:**
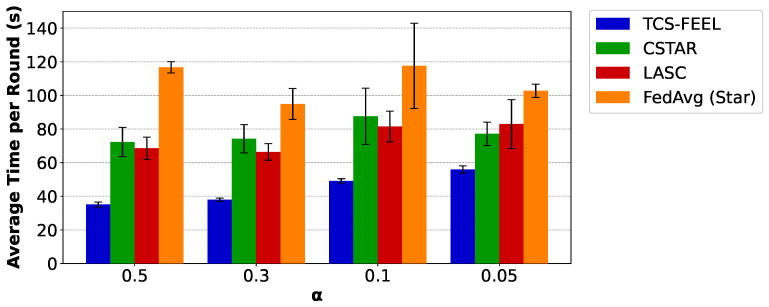
The average time per round for our method and baseline schemes on the CIFAR-10 dataset under different non-IID settings (runs n=5, mean ± std).

Similarly, Figure 8 presents the results on the CIFAR-10 dataset. Consistent with the observations on MNIST, when α=0.5, the proposed tree-based topology reduces the per-round latency by 51.4% compared with CSTAR (p<0.01), by 48.8% compared with LASC (p<0.05), and by 69.9% compared with FedAvg (Star) (p<0.01). When α=0.3, TCS-FEEL reduces the per-round latency by 48.9% compared with CSTAR (p<0.01), by 42.8% compared with LASC (p<0.05), and by 60.0% compared with FedAvg (Star) (p<0.01). When α=0.1, TCS-FEEL reduces the per-round latency by 43.9% compared with CSTAR (p<0.05), by 39.7% compared with LASC (p<0.05), and by 58.2% compared with FedAvg (Star) (p<0.01). When α=0.05, TCS-FEEL reduces the per-round latency by 27.6% compared with CSTAR, by 32.6% compared with LASC (p<0.05), and by 45.6% compared with FedAvg (Star) (p<0.05).

These results demonstrate that our design not only accelerates convergence in terms of rounds but also significantly shortens the time of each training round, leading to faster FL training.

### 6.4. Ablation Study

To evaluate the contribution of individual components in the proposed framework, we compared four schemes: TCS-FEEL, TCS-FEEL (Star), FedAvg (Tree), and FedAvg (Star). The ablation experiments were conducted on both the MNIST and CIFAR-10 datasets under various non-IID levels.

**Figure 9:**
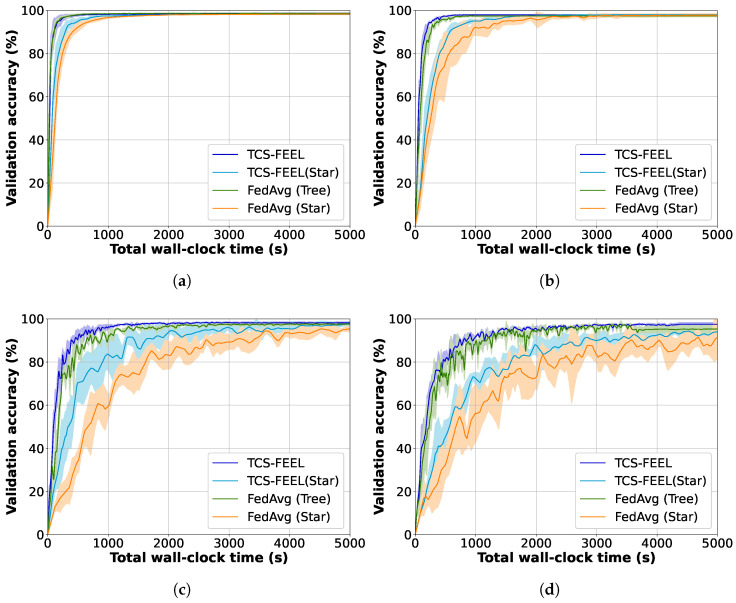
Ablation study on the impact of data heterogeneity (non-IID level α) on model convergence and wall-clock training time using the MNIST dataset (runs n=5): (**a**) α=0.5, (**b**) α=0.1, (**c**) α=0.05, and (**d**) α=0.03.

**Figure 10:**
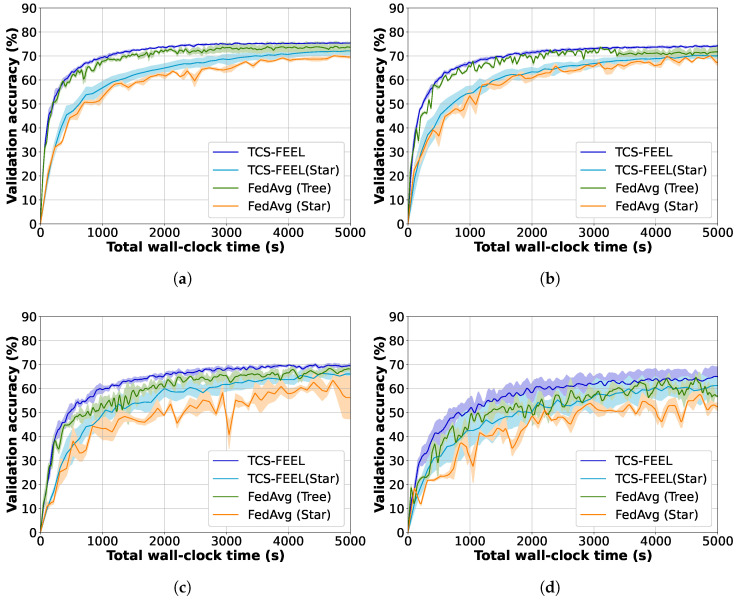
Ablation study on the impact of data heterogeneity (non-IID level α) on model convergence and wall-clock training time using the CIFAR-10 dataset (runs n=5): (**a**) α=0.5, (**b**) α=0.3, (**c**) α=0.1, and (**d**) α=0.05.

As shown in Figure 9 and Figure 10, data heterogeneity significantly influences model convergence and training efficiency. When α decreases, the convergence speed of all methods decreases. Nevertheless, TCS-FEEL consistently achieves higher accuracy and shorter wall-clock training time across all α values, demonstrating robustness under highly heterogeneous conditions.

**Figure 11:**
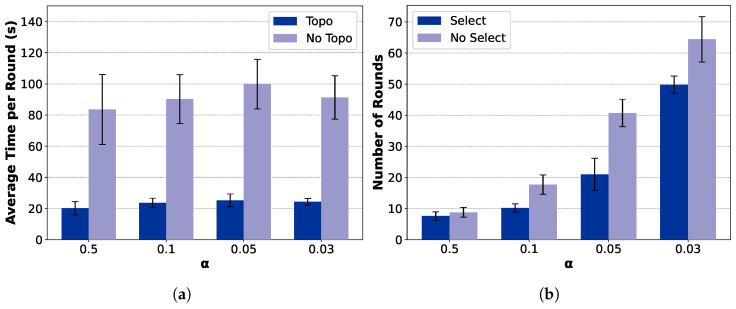
Ablation study on MNIST dataset (runs n=10, mean ± std): (**a**) comparison between topology-aware and topology-free settings in terms of wall-clock training time and (**b**) comparison between selection-based and non-selection schemes in terms of convergence speed.

**Figure 12:**
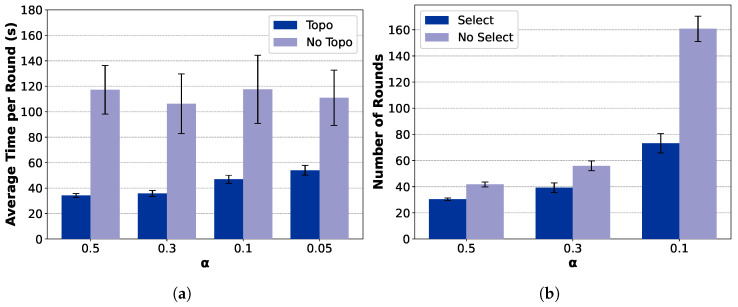
Ablation study on CIFAR-10 dataset (runs n=10, mean ± std): (**a**) comparison between topology-aware and topology-free settings in terms of wall-clock training time and (**b**) comparison between selection-based and non-selection schemes in terms of convergence speed.

Figure 11 and Figure 12 further analyze the contribution of the key modules, including the topology construction and client selection mechanisms. Among them, TCS-FEEL and FedAvg (Tree) adopt a tree-based topology construction mechanism (*Topo*), while the remaining two employ a star-shaped network (*No Topo*). Similarly, TCS-FEEL and TCS-FEEL (Star) include the client selection module (*Select*), whereas the other two baselines operate without selection (*No Select*).

**Impact of Network Topology Optimization:** As illustrated in Figure 11a and Figure 12a, the proposed tree-based topology significantly reduces the per-round latency compared with the star topology. The topology construction module yields over 75.4% lower per-round latency on the MNIST dataset and 66.4% lower latency on the CIFAR-10 dataset (p<0.05). This confirms that adaptive topology formation effectively mitigates communication bottlenecks by enabling multi-hop routing and D2D relaying, thereby accelerating aggregation and enhancing scalability in dynamic edge networks.

**Impact of Client Selection:** As shown in Figure 11b and Figure 12b, the client selection module further accelerates model convergence by prioritizing high-quality and well-connected clients, particularly under highly non-IID conditions. Specifically, when α=0.05, it reduces the number of convergence rounds by 39.8% on MNIST (p<0.05) and, when α=0.1, by 55.3% on CIFAR-10 (p<0.01). This demonstrates that intelligent selection not only shortens the convergence process but also complements topology adaptation in minimizing total wall-clock time.

Overall, both components contribute distinct yet complementary benefits. Topology optimization reduces intra-round latency by improving connectivity, while client selection decreases the number of rounds required for convergence. Their integration in TCS-FEEL leads to substantial end-to-end performance improvements, achieving superior scalability and robustness across diverse non-IID scenarios.

### 6.5. Control-Plane Overhead Analysis

In addition to evaluating the per-round latency from the data plane, we further analyzed the control-plane overhead, which primarily includes the shortest-path computation (Algorithm 1) and the client selection procedure (Algorithm 2) executed in each training round. The CPU execution time required for these two operations for different numbers of clients is summarized in Table 5. The implementation for measuring the control-plane overhead is provided in the Supplementary Material (benchmark_control_plane.py).

The results reveal that the control-plane overhead grows sublinearly with the number of clients, which aligns with the theoretical complexity of the underlying algorithms. Importantly, even at a large scale with N=10,000 clients, the total control-plane overhead remains below 0.1 s per round, whereas the average time per round for local training and communication typically ranges from several tens to hundreds of seconds.

This comparison demonstrates that the optimization cost introduced by our control plane is negligible relative to the overall per-round latency. Therefore, the proposed TCS-FEEL framework maintains scalability and efficiency even in large-scale FL deployment, and the control procedures do not become the bottleneck of the system.

### 6.6. Energy Efficiency Analysis

In this section, we evaluate the communication energy consumption of the proposed TCS-FEEL framework and compare it against two baseline topologies: STAR, where each selected UD directly uploads to the BS without D2D links, and CSTAR, where only the selected UDs can serve as relays. To evaluate the energy efficiency of the proposed framework, we model the per-client communication energy consumption by decomposing it into two components: transmission energy and relay energy.

The transmission energy $E_{\mathrm{comm}(\mathrm{self})}$ represents the energy consumed by a UD when transmitting model parameters either directly to the BS or to a neighboring node in a D2D link. It is given by

(28) $$E_{\mathrm{comm}(\mathrm{self})}=P_{\mathrm{tx}}\cdot T_{\mathrm{tx}},$$

where $P_{\mathrm{tx}}$ is the transmission power of the UD and $T_{\mathrm{tx}}$ denotes the total transmission time. The transmission time $T_{\mathrm{tx}}$ depends on the model size *M* and the achievable throughput $\gamma_{\mathrm{tx}}$ and is given by

(29) $$T_{\mathrm{tx}}=\frac{M}{\gamma_{\mathrm{tx}}},$$

where $\gamma_{\mathrm{tx}}$ is determined by the Shannon’s capacity of the wireless link, as expressed in Equation (27).

The relay energy $E_{\mathrm{comm}(\mathrm{relay})}$ accounts for the additional energy consumed by intermediate UDs that receive and forward data during multi-hop D2D communication. It is defined as

(30) $$E_{\mathrm{comm}(\mathrm{relay})}=P_{\mathrm{rx}}\cdot T_{\mathrm{rx}}+P_{\mathrm{tx}}\cdot T_{\mathrm{fwd}},$$

where $P_{\mathrm{rx}}$ and $P_{\mathrm{tx}}$ denote the reception and transmission power of the relay device, respectively. Here, $T_{\mathrm{rx}}$ is the total reception time required for the relay to receive the model update from the previous hop, and $T_{\mathrm{fwd}}$ is the forwarding transmission time needed to forward the received data to the next hop. Both $T_{\mathrm{rx}}$ and $T_{\mathrm{fwd}}$ are determined by the model size and the achievable throughput of the corresponding D2D links. Specifically, they can be expressed as

(31) $$T_{\mathrm{rx}}=\frac{M}{\gamma_{\mathrm{rx}}},\;T_{\mathrm{fwd}}=\frac{M}{\gamma_{\mathrm{fwd}}},$$

where $\gamma_{\mathrm{rx}}$ denotes the achievable throughput of the incoming D2D link and $\gamma_{\mathrm{fwd}}$ denotes the achievable throughput of the outgoing D2D link at the relay.

Finally, the total communication energy consumption $E_{\mathrm{comm}(\mathrm{total})}$ per client is the sum of the transmission and relay components:

(32) $$E_{\mathrm{comm}(\mathrm{total})}=E_{\mathrm{comm}(\mathrm{self})}+E_{\mathrm{comm}(\mathrm{relay})}.$$

For communication energy modeling, the transmit and receive power of UDs are set to $P_{\mathrm{tx}}=1.0\;W$ and $P_{\mathrm{rx}}=0.2\;W$, respectively. These values are commonly adopted in prior studies [33] and reflect the typical power levels of short-range radios operating in urban and vehicular scenarios. To ensure a fair comparison across all schemes, in each training round, 10% of the UDs are randomly selected to participate in local training. For the energy consumption metrics, we report the average values computed over all UDs, including those not selected as active clients.

Table 6 presents the per-client average transmission energy ($E_{\mathrm{comm}(\mathrm{self})}$), relay energy ($E_{\mathrm{comm}(\mathrm{relay})}$), and total communication energy ($E_{\mathrm{comm}(\mathrm{total})}$) under the three schemes. The results are obtained by averaging over 20 independent simulation runs with N=100 UDs. From the results, we observe that the proposed TCS-FEEL achieves the lowest total communication energy consumption among the three schemes. Specifically, the total per-client communication energy is reduced from 0.1145 J in the STAR topology to 0.0854 J in TCS-FEEL, corresponding to a reduction of approximately 25.4%. This improvement arises from the utilization of multi-hop D2D relaying, which shortens the transmission distance and reduces transmission power while maintaining efficient data delivery. Although the relay energy consumption slightly increases compared with STAR, the significant decrease in direct transmission energy leads to a lower overall energy cost.

Furthermore, TCS-FEEL outperforms CSTAR by leveraging the forwarding capability of all participating clients, instead of restricting relaying to selected UDs only. This broader relay participation improves energy efficiency while ensuring robust data delivery paths in dynamic topologies. These results confirm that D2D-assisted topology-aware client selection not only enhances model convergence but also achieves superior energy efficiency in federated edge learning systems.

### 6.7. Sensitivity Analysis Under Bandwidth Scaling

To evaluate the robustness of TCS-FEEL under more realistic communication conditions, we performed a sensitivity analysis that relaxes the orthogonal-link assumption for D2D communication. In the baseline configuration, all D2D and D2S links were assumed to be orthogonal; i.e., each link operated on an independent frequency band without interference. To approximate bandwidth sharing and contention among concurrent D2D links, we introduced an effective bandwidth scaling factor η∈{1.0,0.75,0.5,0.25}, where the effective bandwidth for each D2D link is given by

$$B_{\mathrm{eff}}^{D2D}=\eta B,$$

with *B* being the nominal channel bandwidth. The D2S links were kept orthogonal and unaffected by bandwidth scaling, maintaining their original bandwidth *B*.

When η decreases, only the D2D links experience proportionally reduced transmission rates, emulating the impact of spectrum reuse or interference-limited D2D communication. The per-round communication latency $T_{\mathrm{comm}}$ was then recalculated as

$$T_{\mathrm{comm}}=\sum_{\ell \in L_{D2S}}\frac{{\mathrm{payload}}_{\ell }}{B\log_{2}\left(1+{\mathrm{SNR}}_{\ell }\right)}+\sum_{\ell \in L_{D2D}}\frac{{\mathrm{payload}}_{\ell }}{B_{\mathrm{eff}}^{D2D}\log_{2}\left(1+{\mathrm{SNR}}_{\ell }\right)},$$

where $L_{D2S}$ and $L_{D2D}$ denote the sets of D2S and D2D links, respectively. The rest of the training and aggregation pipeline remained unchanged, and each experiment was repeated five times with different random seeds to ensure statistical reliability.

**Figure 13:**
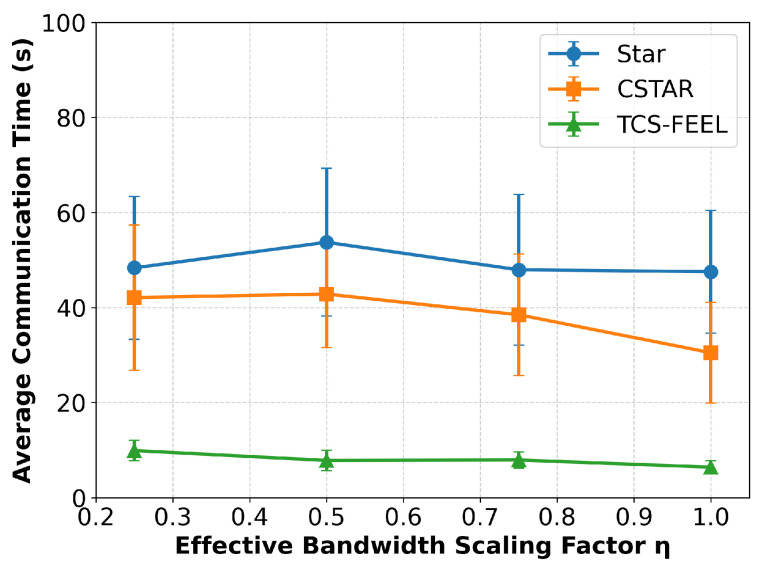
Sensitivity analysis of per-round communication latency under different effective bandwidth scaling factors η (runs n=5, mean ± std). Lower η values represent stronger bandwidth contention among D2D links.

As illustrated in Figure 13, the overall per-round communication time of both TCS-FEEL and CSTAR increases as the effective bandwidth scaling factor η decreases, which is expected since a lower η limits the available transmission capacity. When η=0.5, TCS-FEEL achieves 75.6% and 81.4% lower per-round communication time than CSTAR and FedAvg (Star), respectively (p<0.01). Even under the most constrained condition (η=0.25), TCS-FEEL still maintains substantial advantages, achieving 71.4% and 73.8% lower per-round communication time compared with CSTAR and FedAvg (Star), respectively (p<0.01).

### 6.8. Topological Dynamics Analysis

To evaluate the robustness of the proposed TCS-FEEL framework under device mobility, we analyze the topological dynamics of the D2D communication graph over time using the link change ratio (LCR). The LCR is defined as the proportion of links that differ from the initial topology and serves as an indicator of topology stability in dynamic environments. A higher LCR indicates more frequent changes in network topology. Formally, the LCR at time *t* is expressed as

(33) $$\mathrm{LCR}\left(t\right)=\frac{\left|E(t)▵E(0)\right|}{\left|E(0)\right|},$$

where E(0) denotes the set of communication links in the initial topology at t=0, E(t) is the set of links at time *t*, and Δ represents the symmetric difference between the two sets. A higher LCR indicates that a larger fraction of links have changed compared with the initial topology.

We simulate N=100 UDs with random initial positions and evaluate three time snapshots t∈{20,40,60} s relative to the baseline at t=0 s, at three different mobility speeds: v=1m/s, 5m/s, and 10m/s. In each round, 10% of the UDs are randomly selected as participating clients for a fair comparison. We compare the LCR evolution for our TCS-FEEL and CSTAR.

**Figure 14:**
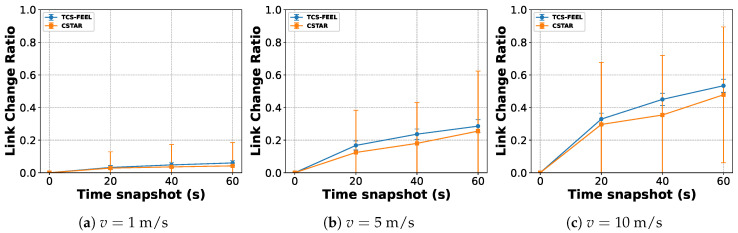
Link change ratio evolution at different mobility speeds (runs n=5, mean ± std): (**a**) 1m/s, (**b**) 5m/s, and (**c**) 10m/s.

Figure 14 shows the evolution of the LCR for both methods. As expected, the LCR increases with mobility speed due to more frequent link formation and breakage events. It is important to note that a higher LCR itself does not directly indicate greater robustness; rather, it reflects a more dynamic communication environment that poses greater challenges for maintaining stable connectivity and low latency. However, TCS-FEEL consistently exhibits a higher LCR than CSTAR across all speeds and time snapshots while still maintaining stable communication performance, demonstrating its stronger robustness against topology dynamics. This is because allowing all UDs to participate in relaying provides more redundant communication paths, thereby mitigating the impact of node mobility on network connectivity.

## 7. Discussion

This section discusses the comparative performance and robustness of the proposed TCS-FEEL framework. We first compare TCS-FEEL with representative communication-efficient FL approaches to highlight its advantages in dynamic edge environments. We then examine its robustness when relaxing modeling assumptions such as link orthogonality, showing that the proposed framework maintains stable performance even under bandwidth-limited and interference-prone conditions.

### 7.1. Comparison with Related Works

While numerous studies have investigated communication-efficient FL, most differ from our work in assumptions, problem settings, or design objectives. For instance, hierarchical FL approaches such as [16,17,18,19] assume static two-tier structures with fixed relay nodes, which fail to capture the rapid topology variations caused by device mobility in edge environments. Similarly, deep learning-based topology optimization [20] relies on global topology collection for training, introducing high latency and making it impractical for dynamic edge scenarios.

Chen et al. [28] proposed a latency-aware client selection framework (LACS) that jointly optimizes client participation probabilities and total training rounds to mitigate system heterogeneity. While effective under static network conditions, LACS performs offline optimization and assumes fixed links, preventing real-time adaptation to mobility or fluctuating link quality. In contrast, our TCS-FEEL performs online topology adaptation and stochastic client selection in each round, allowing it to dynamically respond to topology and bandwidth variations.

Experimental results strongly validate the effectiveness of this design. As shown in Figure 4 and Table 3, TCS-FEEL achieves the fastest convergence on MNIST across all non-IID settings. When α=0.1, it reduces convergence rounds by 28.2% compared with CSTAR and by more than 42.0% compared with LACS and FedAvg (Star); at α=0.05, these reductions reach 29.1% and 45.9% (p<0.05), respectively. On the CIFAR-10 dataset (Figure 5, Table 4), TCS-FEEL achieves up to 49.7% fewer rounds than FedAvg (Star) and maintains comparable or better performance than LACS under moderate heterogeneity (α=0.1). These results confirm that jointly optimizing topology and client selection effectively accelerates convergence under heterogeneous and dynamic conditions.

In terms of per-round latency, Figure 7 and Figure 8 further demonstrate that TCS-FEEL consistently outperforms existing methods. On MNIST, when α=0.1, TCS-FEEL reduces per-round latency by 57.3% compared with CSTAR, by 63.6% compared with LACS, and by 72.0% compared with FedAvg (Star) (p<0.01). On CIFAR-10, the reductions reach 43.9%, 39.7%, and 58.2%, respectively (p<0.05). These improvements arise because TCS-FEEL’s dynamic tree-based topology enables multi-hop D2D relaying and adaptive link selection, reducing bottlenecks inherent in the star topology and improving bandwidth utilization.

Overall, compared with CSTAR, LACS, and FedAvg (Star), TCS-FEEL advances the state of the art in topology- and client selection-aware FL. By integrating adaptive topology construction with stochastic client selection, TCS-FEEL achieves both faster convergence (a reduction in rounds of up to 49.7%) and lower communication latency (a reduction of up to 77.3% per round) across diverse datasets and non-IID levels. These results highlight its robustness and scalability for real-world mobile edge learning scenarios.

### 7.2. Discussion on Interference and Robustness

It is important to note that our throughput model initially assumed orthogonal spectrum allocation and neglected inter-link interference for analytical tractability. To examine the robustness of this assumption, we conducted a sensitivity analysis under bandwidth scaling, where only D2D links experienced effective bandwidth reduction to emulate interference and spectrum contention effects.

As shown in Figure 13, the per-round latency of CSTAR increases markedly as the effective bandwidth scaling factor η decreases, whereas the increase for TCS-FEEL remains relatively minor. Even under the most constrained condition (η=0.25), TCS-FEEL consistently outperforms all baseline methods, with statistically significant gains (p<0.01). These results demonstrate that the communication efficiency advantages introduced by the topology construction and client selection modules remain robust even when the orthogonal-link assumption is relaxed.

Therefore, although the original analytical model may slightly overestimate the absolute throughput, the relative performance superiority of TCS-FEEL persists in interference-limited or bandwidth-constrained scenarios. Incorporating interference-aware scheduling and adaptive spectrum allocation into TCS-FEEL represents a promising direction for future research, further enhancing its scalability and practicality in dense wireless environments.

## 8. Conclusions

This paper presented TCS-FEEL, a novel FL framework tailored to dynamic edge networks, which integrates stochastic client selection with adaptive network topology optimization. The proposed approach jointly addresses both system and statistical heterogeneity. Extensive simulations on widely used datasets under diverse non-IID scenarios demonstrate that TCS-FEEL consistently outperforms existing baselines. Specifically, it requires fewer communication rounds to reach target accuracy levels and significantly reduces the per-round wall-clock time, thereby accelerating global model convergence. Moreover, these improvements become more pronounced under severe data heterogeneity, underscoring the robustness and scalability of the framework. TCS-FEEL provides an intelligent and privacy-preserving solution for sensor-driven edge networks, enabling efficient FL in latency-sensitive and mobility-intensive applications such as autonomous driving, smart city sensing, and the Industrial IoT.

In future work, we aim to broaden the applicability and impact of TCS-FEEL in several directions. First, we plan to integrate advanced privacy-preserving techniques, such as differential privacy and secure aggregation, to further enhance data confidentiality. Second, we will investigate adaptive spectrum sharing and interference-aware communication models to bridge the gap between the current orthogonal bandwidth assumption and real-world wireless environments. Finally, we plan to evaluate TCS-FEEL in real-world testbeds and cross-domain applications, paving the way for its large-scale deployment in sensor-rich edge networks.

## Figures and Tables

**Table 1:** Key notations and definitions.

Symbol	Definition
N	Set of UDs, indexed as {1,…,N}
$D_{i}$	Local dataset on client *i* with size $|D_{i}|$
$x_{i}^{\left(t\right)},y_{i}^{\left(t\right)}$	Feature and label of the *t*-th local sample on client *i*
w	Global model parameters
$F_{i}\left(w\right)$	Empirical local loss of client *i*
F(w)	Global loss as a weighted sum of local losses
$p_{i}$	Relative data weight of client *i* in the global dataset
$w_{i}^{\left((k+1)E\right)}$	Local model after *E* epochs of training in round *k*
${\tilde{w}}^{\left(kE\right)}$	Global model before round *k*
$\varphi_{i}^{\left(k\right)}$	Sampling probability of client *i* in round *k*
$q_{i}^{\left(k\right)}$	Conditional inclusion probability of client *i*
$S_{k}$	Set of clients selected in round *k*
$\Phi_{\mathrm{UL}},\Phi_{\mathrm{DL}},\Phi_{D2D}$	Bandwidth allocated to uplink, downlink, and D2D links
$\gamma_{i,j}^{\left(k\right)}$	Throughput of link (i,j) in round *k*
*M*	Model size in bits
$l^{\left(k\right)}\left(i,j\right)$	Transmission latency on link (i,j) in round *k*
$G^{\left(k\right)}$	Directed graph representation of network in round *k*
$\tau_{i}^{\left(k\right)}$	Average training time per sample on client *i* in round *k*
$T_{i,\mathrm{train}}^{\left(k\right)}$	Local training time for client *i* in round *k*
$T_{↑}^{\left(k\right)}\left(i\right),T_{↓}^{\left(k\right)}\left(i\right)$	Model upload and download times for client *i*
$T^{\left(k\right)}$	Round time defined by the slowest participating client
*E*	Number of local training epochs in each FL round

**Table 2:** Experimental parameters and implementation details.

Parameter	Definition	Value
*N*	Number of UDs (clients)	100
*v*	Average speed of UDs	1,5,10m/s
$\Phi_{D2D}$	Bandwidth of D2D links	50MHz
$\Phi_{\mathrm{UL}}$	Bandwidth of uplink (UD → BS)	10MHz
$\Phi_{\mathrm{DL}}$	Bandwidth of downlink (BS → UD)	10MHz
*C*	Channel capacity utilization rate	0.8
$n_{0}$	Background noise power	−100dBm
$\pi_{i}$	Transmission power of UD *i*	100mW
ζ	Path-loss exponent	3.5
$\tau_{i}^{\left(k\right)}$	Average processing time per sample on UD *i* in round *k*	0.01–0.03s
*M*	Model size	25.09Mb
*E*	Number of local training epochs per round	5

**Table 3:** Number of rounds required to achieve 95% accuracy on MNIST under different non-IID settings (runs n=5, mean ± std).

Method	α=0.5	α=0.1	α=0.05	α=0.03
TCS-FEEL	7.6 ± 1.3	10.2 ± 1.3	21.0 ± 5.1	49.8 ± 2.8
CSTAR	8.4 ± 1.5	14.2 ± 2.6	29.6 ± 3.2	56.0 ± 12.9
LASC	8.6 ± 1.3	17.8 ± 2.4	42.6 ± 4.9	65.2 ± 7.2
FedAvg (Star)	9.0 ± 1.9	17.6 ± 4.0	38.8 ± 3.3	63.6 ± 8.2

**Table 4:** Number of rounds required to achieve 70% accuracy on CIFAR-10 under different non-IID settings (runs n=5, mean ± std).

Method	α=0.5	α=0.3	α=0.1	α=0.05
TCS-FEEL	30.4 ± 0.9	39.2 ± 3.7	72.2 ± 8.2	N.A.
CSTAR	31.2 ± 1.3	38.0 ± 2.8	79.3 ± 5.9	N.A.
LASC	30.8 ± 1.8	38.8 ± 4.1	71.5 ± 2.4	N.A.
FedAvg (Star)	41.2 ± 1.6	55.8 ± 4.0	159.5 ± 11.2	N.A.

**Table 5:** Control-plane overhead per round for different numbers of clients (milliseconds, runs n=5, mean ± std).

Number of Clients *N*	Shortest-Path Computation	Client Selection
100	0.338±0.019	2.059±0.037
1000	3.859±0.128	3.569±0.020
10,000	49.102±1.709	16.337±0.762

**Table 6:** Per-client average communication energy consumption (Joule, runs n=5, mean ± std).

Scheme	$E_{\mathrm{comm}(\mathrm{self})}$	$E_{\mathrm{comm}(\mathrm{relay})}$	$E_{\mathrm{comm}(\mathrm{total})}$
STAR	0.114538±0.109935	0.000000±0.000000	0.114538±0.109935
CSTAR	0.084409±0.041974	0.008048±0.007131	0.092457±0.041948
TCS-FEEL	0.046958±0.004776	0.038485±0.005191	0.085443±0.007634

## Data Availability

The data presented in this article are available upon request from the corresponding author.

## Supplementary Materials

The following supporting information can be downloaded at: https://www.mdpi.com/article/10.3390/s25216534/s1.
